# Effectiveness of Omega-3 Polyunsaturated Fatty Acids in Non-alcoholic Fatty Liver Disease: A Systematic Review and Meta-Analysis

**DOI:** 10.7759/cureus.68002

**Published:** 2024-08-28

**Authors:** Tarique Aziz, Mukesh K Niraj, Shishir Kumar, Rajendra Kumar, Hina Parveen

**Affiliations:** 1 Biochemistry, Rajendra Institute of Medical Sciences, Ranchi, IND; 2 Physiology, Rajendra Institute of Medical Sciences, Ranchi, IND; 3 Biochemistry, King George’s Medical University, Lucknow, IND

**Keywords:** non-alcoholic steatohepatitis, non-alcoholic fatty liver disease (nafld), meta-analysis, gamma-glutamyl transferase, omega-3 polyunsaturated fatty acids

## Abstract

Non-alcoholic fatty liver disease (NAFLD) is a prevalent liver disorder characterized by excessive hepatic fat accumulation without alcohol intake. It can progress to non-alcoholic steatohepatitis, increasing the risk of cirrhosis and liver failure. This study aims to evaluate the efficacy of omega-3 polyunsaturated fatty acids (n-3 PUFAs) in treating NAFLD. A systematic review and meta-analysis was conducted including studies published from January 2018 to June 2023. Databases searched included PubMed, Embase, Cochrane Library, and ClinicalTrials.gov. Inclusion criteria comprised randomized controlled trials and cohort studies involving human subjects or animal models with NAFLD. Data were extracted and analyzed to assess the impact of omega-3 PUFAs on liver fat, hepatic enzymes, and serum lipid profiles using RevMan 5.4. A total of 15 studies met the inclusion criteria. Omega-3 supplementation significantly decreased alanine aminotransferase (ALT) (mean difference = -2.12, 95% confidence interval (CI) = -3.36, -0.87) and aspartate aminotransferase (AST) (mean difference = -1.50, 95% CI = -2.59, -0.42). Gamma-glutamyl transferase levels showed a trend toward reduction (mean difference = -0.82, 95% CI = -1.66, 0.02). Serum lipid profiles improved significantly with reductions in triglycerides, low-density lipoprotein, and total cholesterol along with significant reductions in AST, ALT, and alkaline phosphatase in animal models. Omega-3 PUFAs appear to offer beneficial effects on liver enzymes, serum lipid profiles, and anthropometric indices in NAFLD patients. While their impact on liver fat content remains uncertain, omega-3 supplementation could serve as a valuable adjunct treatment for enhancing metabolic profiles and liver function in NAFLD patients.

## Introduction and background

Non-alcoholic fatty liver disease (NAFLD) is characterized by the accumulation of fat in more than 5% of hepatocytes, occurring without significant alcohol intake [1]. It is a prevalent condition globally, affecting 10-24% of the population, with even higher rates observed in Western nations and some regions of China, where the prevalence ranges from 1% to 35% for NAFLD and 15% to 39% for non-alcoholic steatohepatitis (NASH) [2,3]. NASH, a severe form of NAFLD, can progress to cirrhosis in 20% of cases, with one-third of patients developing advanced fibrosis [4]. The pathophysiology of NAFLD is complex, involving factors such as excessive dietary fat intake, peripheral insulin resistance, oxidative stress, and innate immunity [5]. It is also associated with various metabolic conditions, including cardiovascular disease, dyslipidemia, obesity, metabolic syndrome, and type 2 diabetes mellitus (T2DM) [6]. Current treatment approaches for NAFLD/NASH focus on lifestyle interventions, aiming for a 7-10% weight loss, which has been shown to improve liver enzymes and histology. However, no medications have been officially approved for NASH treatment, despite some having undergone phase III trials, including lipid-lowering agents such as n-3 polyunsaturated fatty acids (3PUFA), antioxidants such as vitamin E, and insulin sensitizers such as metformin and thiazolidinediones [7-9].

Omega-3 PUFAs include docosapentaenoic acid (DPA), eicosapentaenoic acid (EPA), stearidonic acid (SDA), docosahexaenoic acid (DHA), and alpha-linolenic acid (α-ALA), all of which are essential fatty acids [10]. Sources of α-ALA include seeds, nuts, and vegetable oils, while marine animals primarily provide DPA, with DHA and EPA being the main n-3 PUFAs in fish oils [11]. EPA and DHA are known to significantly reduce very low-density lipoproteins (VLDLs), which transform into low-density lipoprotein (LDL), intermediate-density lipoprotein (IDL), and triglycerides (TGs), thereby potentially preventing coronary artery disease [12]. A dietary imbalance with excessive omega-6 PUFAs and insufficient omega-3 PUFAs is believed to contribute to NAFLD [13]. Studies indicate that NAFLD patients have a higher n-6/n-3 ratio and a lower PUFA content [14]. Omega-3 PUFAs have shown beneficial effects on hypertension, hyperlipidemia, endothelial dysfunction, and cardiovascular disease, acting as negative regulators of hepatic lipogenesis and inflammation [15].

Previous meta-analyses have demonstrated positive effects of omega-3 PUFAs on lipid profiles, liver fat, and certain liver enzymes in NAFLD patients [16,17]. Lu et al. found improvements in gamma-glutamyl transferase (GGT) and liver fat, but not in alanine aminotransferase (ALT), aspartate aminotransferase (AST), total cholesterol (TC), or LDL [16]. Lee et al. reported that omega-3 PUFAs significantly reduced liver fat and improved TGs, TC, high-density lipoprotein (HDL), and body mass index (BMI) [17]. However, these meta-analyses may not reflect the most current evidence, with one relying solely on randomized controlled trials (RCTs). To address this gap, our meta-analysis aims to provide the latest, comprehensive evidence on the efficacy of omega-3 PUFAs in treating NAFLD.

## Review

Methodology

Literature Search

The search strategy was designed to identify studies assessing the impact of omega-3 PUFAs and *Phaleria macrocarpa* on NAFLD. A comprehensive search was conducted across multiple databases, including PubMed, Embase, Cochrane Library, and ClinicalTrials.gov, using search terms such as “non-alcoholic fatty liver disease,” “NAFLD,” “omega-3 fatty acids,” “n-3 PUFAs,” “Phaleria macrocarpa,” and “liver fat,” combined with Boolean operators (AND, OR).

Selection and Eligibility Criteria

The inclusion criteria comprised studies investigating the impact of omega-3 PUFAs or *Phaleria macrocarpa *on NAFLD, specifically RCTs and cohort studies, articles published in English, studies involving human subjects or animal models with NAFLD, and articles from peer-reviewed journals with clear methodologies and relevant data, published between January 2018 and June 2023. Exclusion criteria included review articles, editorials, letters, conference abstracts, studies not specifically focusing on NAFLD or not assessing the effects of omega-3 PUFAs, non-English-language articles, and studies published before 2018. Studies were identified through a systematic search of electronic databases, with titles and abstracts of identified studies screened for relevance. Full texts of potentially eligible studies were reviewed to confirm eligibility based on the inclusion and exclusion criteria. The final selection included studies that met all criteria and provided comprehensive data on the effects of the interventions. A total of 15 studies were included in the final analysis, representing a diverse range of participant populations and study designs.

Data Extraction and Quality Assessment

The methodological quality of the included studies was evaluated using appropriate tools, with the Cochrane Collaboration’s risk of bias tool utilized for RCTs. Quality assessment was performed independently by two reviewers, with any discrepancies resolved through discussion or consultation with a third reviewer if needed. Data were extracted from selected studies using a standardized form, with key information including study characteristics, study design, participant demographics, intervention details, and outcomes related to liver fat, hepatic enzymes, serum lipid profiles, and other relevant measures systematically recorded and verified for accuracy.

Statistical Analysis

A meta-analysis was conducted to synthesize the quantitative data from the included studies. Effect sizes, such as mean differences, were calculated for continuous outcomes. Statistical heterogeneity among studies was assessed using the I^2^ statistic, with values greater than 50% indicating substantial heterogeneity. Random-effect models were employed to account for variability between studies. Sensitivity analyses and subgroup analyses were conducted to explore sources of heterogeneity and assess the robustness of the findings using the Review Manager (RevMan) software version 5.4 (RevMan 5; The Nordic Cochrane Centre, The Cochrane Collaboration, Copenhagen, Denmark). The significance level was set at p-values <0.05.

Results

Study Selection

**Figure 1:**
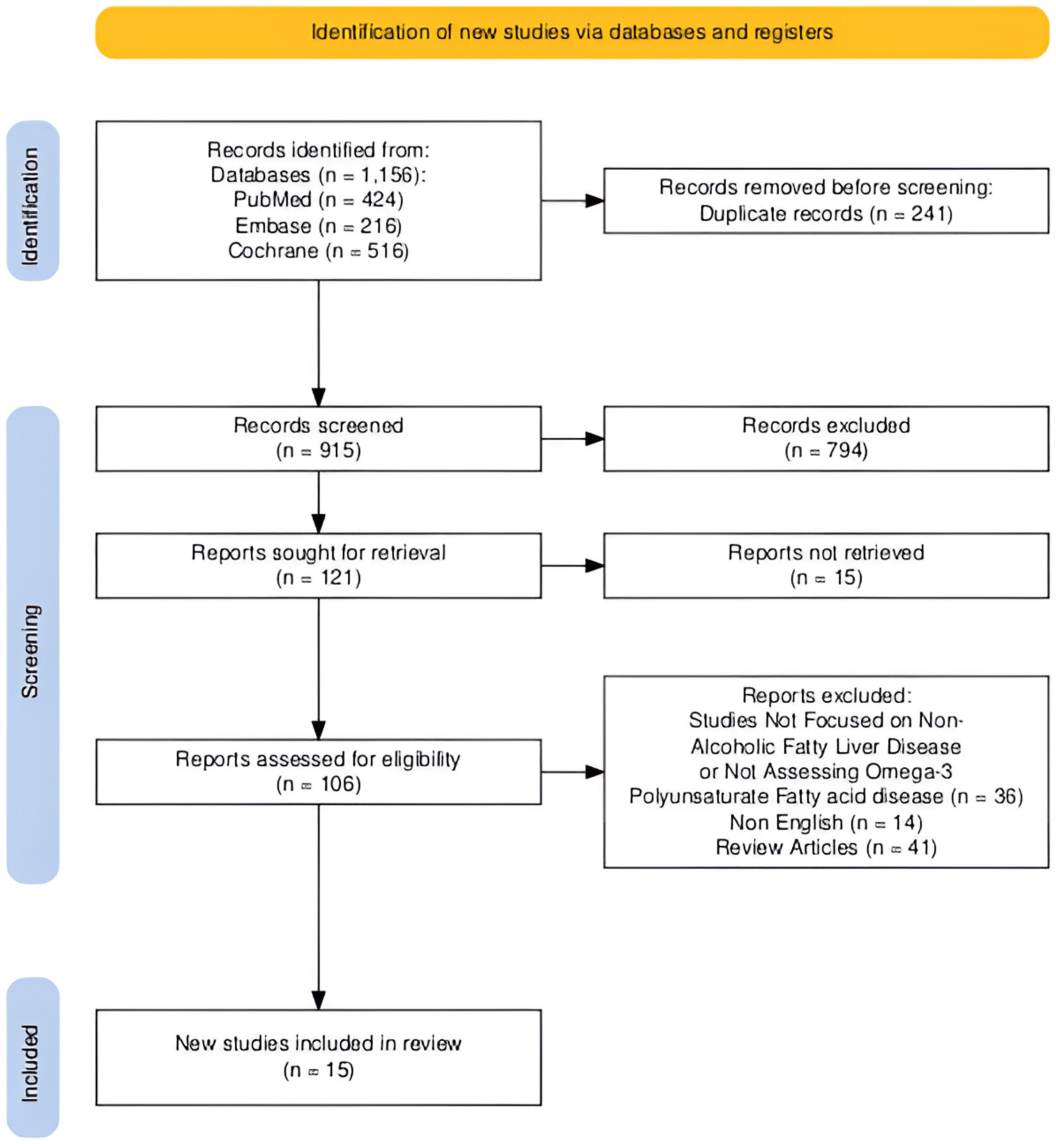
Preferred Reporting Items for Systematic Reviews and Meta-Analyses (PRISMA) flow diagram.

The initial literature search yielded a total of 1,156 publications described using the Preferred Reporting Items for Systematic Reviews and Meta-Analyses (PRISMA) diagram [18]. After a meticulous assessment of abstracts and titles, 794 articles were excluded, and 15 were not retrieved. In total, 106 articles were deemed relevant, and their full texts were acquired for further examination. A total of 91 studies that did not fulfill the inclusion criteria or did not explicitly investigate the accuracy of omega-3 fatty acids in the treatment of NAFLD were excluded. Following a thorough screening process, 15 studies were found to be appropriate for inclusion in this systematic review and meta-analysis (Figure 1).

Study Characteristics

The systematic review included 15 studies assessing the impact of omega-3 supplementation on liver-related outcomes. This comprised 14 RCTs and one cohort study, showcasing a diverse range of research designs. The study populations included male Sprague-Dawley rats and patients with NAFLD, T2DM, metabolic syndrome, and overweight/obesity. Sample sizes varied widely, ranging from 20 to 176 participants. The duration of treatments ranged from eight to twenty-four weeks, with one cohort study extending over eleven years. Various forms and doses of omega-3 supplementation were used, including pure omega-3 PUFA, combinations with additional supplements such as dapagliflozin or fenofibrate, and comparisons with placebo groups. The outcomes evaluated included numerous liver-related measures such as liver fat content, liver enzymes (ALT, AST), liver volume, liver histology, and other metabolic indicators, including lipid profiles, inflammatory markers, and indices of liver fibrosis (Table 1).

**Table 1:** Characteristics and results of the studies reviewed.

Author	Year	Study design	Population number and type	Intervention	Duration	Outcomes assessed	Results with stats	Conclusions
Vell et al., 2023 [19]	2023	Cohort study	Large cohort of individuals	Omega-3 supplementation	11 years	Incident liver disease, alcoholic liver disease, liver failure, non-alcoholic liver disease (NAFLD)	Hazard ratio (HR) = 0.716; 95% confidence interval (CI) = 0.639, 0.802; p = 7.6 × 10^−9^ for liver disease; HR = 0.559; 95% CI = 0.347, 0.833; p = 4.3 × 10^−3^ for alcoholic liver disease; HR = 0.548; 95% CI = 0.343, 0.875; p = 1.2 × 10^−2^ for liver failure; HR = 0.784; 95% CI = 0.650, 0.944; p = 1.0 × 10^−2^ for non-alcoholic liver disease; HR = 0.846; 95% CI = 0.777, 0.921; p = 1.1 × 10^−4^ for NAFLD	Omega-3 supplementation may reduce the incidence of liver disease. Further evaluation in clinical trials is warranted
Orang et al., 2020 [20]	2020	Randomized double-blind trial	44 adult patients with T2DM and NAFLD	2 g/day omega-3	12 weeks	Beta-cell function, interleukin-6 (IL-6), fasting blood glucose (FBG), HbA1C, serum insulin, C-reactive protein (CRP), tumor necrosis factor-alpha (TNF-α), Homeostatic Model Assessment for Insulin Resistance(HOMA-IR), insulin sensitivity	Significant increase in beta-cell function and IL -6 in omega-3; no effect on other glycemic and insulin resistance parameters or inflammatory markers	Omega-3 supplementation significantly increased beta-cell function and IL-6 but had no effect on other glycemic and inflammatory markers
Farhangi et al., 2022 [21]	2022	Randomized controlled trial	36 NAFLD patients	Resistant dextrin and Camelina sativa oil (RDCSO) vs. Camelina sativa oil (CSO) + maltodextrin	12 weeks	Body mass index (BMI), weight, waist circumference (WC), liver steatosis grade, ALT, ALP, AST/ALT ratio, TC, TG, HDL-c, LDL-c/HDL-c (Castelli II Index), Log TG/HDL-c (athero-index), hs-CRP, leptin, adiponectin (LPS)	Significant improvements in anthropometric indices, liver enzymes, lipid profile, athero-index, adiponectin, metabolic endotoxemia in RDCSO group compared to CSO group	RDCSO co-supplementation is more effective than CSO alone in improving lipid profile, liver enzymes, and other metabolic markers in NAFLD patients
Oscarsson et al., 2018 [22]	2018	Double-blind, randomized, placebo-controlled	78 overweight or obese individuals with NAFLD and hypertriglyceridemia	4 g omega-3 carboxylic acids (OM-3CA), 200 mg fenofibrate, or placebo	12 weeks	Proton density fat fraction (PDFF), liver volume, pancreas volume, adipose tissue volumes	Placebo: +4% liver PDFF, OM-3CA: −2% liver PDFF, Fenofibrate: +17% liver PDFF; fenofibrate increased liver and pancreas volumes significantly	OM-3CA and fenofibrate reduced serum triglycerides but did not reduce liver fat. Fenofibrate increased total liver volume and fat
Sangouni et al., 2021 [23]	2021	Randomized controlled trial	60 diabetic patients with NAFLD	2,000 mg/d omega-3 or placebo	12 weeks	Fatty liver index, lipid accumulation product, visceral adiposity index	Significant improvement in fatty liver index (−3.6 ± 12.1 vs. 0.9 ± 8.9; p = 0.04), lipid accumulation product (−14.2 ± 27.9 vs. 8.0 ± 26.3; p = 0.002), visceral adiposity index (−0.5 ± 0.9 vs. 0.0 ± 0.8; p = 0.01)	Omega-3 supplementation for 12 weeks improves fatty liver index, lipid accumulation product, and visceral adiposity index
Song et al., 2020 [24]	2020	Double-blind placebo-controlled trial	96 NAFLD subjects	PS (3.3 g/d), FO (450 mg EPA + 1,500 mg DHA/d), PS + FO, or placebo		Liver attenuation ratio (L ratio), TGF-β, TNF-α, TAG, total cholesterol	PS + FO group: 36% increase in L ratio (p = 0.083); TGF-β and TNF-α significantly decreased in study groups; TAG and total cholesterol reduced by 11.57% and 9.55%, respectively, in PS + FO group	Co-supplementation of PS and EPA + DHA could increase the effectiveness of treatment for hepatic steatosis
Parker et al., 2019 [25]	2019	Double-blind, randomized controlled trial	50 overweight men (BMI 25.0–29.9 kg/m², waist >94 cm)	Fish oil (1,728 mg marine triglycerides with 588 mg EPA and 412 mg DHA) or placebo (olive oil)	12 weeks	Liver fat, liver tests, body composition (including VAT)	No significant time or group × time effect for liver fat, liver enzymes, anthropometry, or body composition including VAT (p > 0.05 for all)	Omega-3 PUFA did not reduce liver fat in overweight men. Factors determining individual health benefits need clarification
Eriksson et al., 2018 [26]	2018	Double-blind randomized placebo-controlled trial	84 participants with type 2 diabetes and NAFLD	10 mg dapagliflozin, 4 g omega-3 carboxylic acids (OM-3CA), combination, or placebo	12 weeks	Liver PDFF, liver volume, markers of glucose and lipid metabolism, hepatocyte injury, oxidative stress	OM-3CA: −15% liver PDFF, dapagliflozin: −13% liver PDFF, combination: −21% liver PDFF (p = 0.046); combination reduced liver fat volume (−24%, p = 0.037); dapagliflozin reduced hepatocyte injury biomarkers and FGF21	Combined treatment with dapagliflozin and OM-3CA significantly reduced liver fat content. dapagliflozin showed disease-modifying effect in NAFLD
Sundari et al., 2018 [27]	2018	Randomized controlled trial	Male Sprague-Dawley rats (n = 30)	Carbon tetrachloride (CCl4), CCl4 + N-acetylcysteine (NAC), CCl4 + various doses of Phaleria macrocarpa (PM) water extract (50, 100, 150 mg/kg body weight (BW)	8 weeks	AST, ALT, ALP, liver histopathology, MDA, GSH/GSSG ratio, TNF-α, TGF-β1	Water extract and NAC significantly reduced AST, ALT, ALP, MDA, TNF-α, TGF-β1; increased total glutathione (GSH/GSSG) ratio (p < 0.05)	Water extract of Phaleria macrocarpa prevents CCl4-induced liver fibrosis through antioxidant and anti-inflammatory activities
Wardhani et al., 2020 [28]	2020	Randomized controlled trial	Male Sprague-Dawley rats (n = 60)	CCl4, Silymarin (100 mg/kg BW), Phaleria macrocarpa extract (75 or 150 mg/kg BW)	8 weeks	Liver function, liver damage and fibrosis markers, oxidative stress markers, TGF-β1, MMP-13	PM extract and silymarin decreased MDA, TGF-β1, MMP-13, normalized liver function, reduced oxidative stress, decreased hepatic stellate cell activation (p < 0.05)	PM extracts ameliorate CCl4-induced liver fibrosis by reducing oxidative stress and regulating pro-fibrogenic cytokines
Spahis et al., 2018 [29]	2018	Randomized controlled trial	20 young male participants with NAFLD	Omega-3 PUFA (2 g/day)	6 months	FLI, ALT, ALT/AST ratio, lipid profile, carotid intima-media thickness, metabolic and oxidative stress markers, adiponectin	Omega-3 PUFA significantly increased EPA and DHA in RBCs, reduced FLI, ALT, ALT/AST ratio, improved lipid profile and carotid intima-media thickness, reduced metabolic and oxidative stress markers, increased adiponectin	Omega-3 PUFA has beneficial effects on liver steatosis and related metabolic abnormalities in NAFLD
Cansanção et al., 2020 [30]	2020	Double-blind, randomized, placebo-controlled	NAFLD individuals (n = 24)	n-3 PUFA (n = 13) vs. placebo (n = 11)	6 months	miR-122 expression, liver fibrosis, RBC fatty acids, biochemical tests	Increased DHA and omega index in RBC (p = 0.022, p = 0.012), reduced ALP (p = 0.002) and liver fibrosis (p = 0.039) in n-3 PUFA group	n-3 PUFA supplementation reduced ALP and liver fibrosis without altering miR-122 expression in NAFLD patients
Šmíd et al., 2022 [31]	2020	Double-blind, randomized, placebo-controlled	Metabolic syndrome and NAFLD patients (n = 60)	n-3 PUFA (3.6 g/day) vs. placebo	12 months	GGT activity, liver fat, plasma lipidome	Decreased GGT activity (2.03 ± 2.8 vs. 1.43 ± 1.6; p < 0.05), liver fat reduction correlated with weight reduction, beneficial changes in plasma lipid profile (p < 0.005)	n-3 PUFA treatment reduced GGT activity and liver fat in those who lost weight, improved plasma lipid profile
Musazadeh et al., 2022 [32]	2022	Triple-blind, placebo-controlled, randomized	NAFLD patients (n = 46)	Camelina sativa oil (CSO) vs. placebo	12 weeks	Anthropometric indices, lipid profile, liver enzymes, adiponectin	Significant differences in weight, BMI, waist circumference, triglycerides, TC, LDL-C, atherogenic index, ALT, adiponectin (p < 0.046 for all) in CSO group; no significant differences in hip circumference, HDL-C, other liver enzymes	CSO supplementation significantly improved anthropometric indices, ALT, lipid profile, and adiponectin in NAFLD patients
Tobin et al., 2018 [33]	2023	Randomized controlled trial	NAFLD patients (n = 176)	High concentrate omega-3 (n = 87) vs. placebo (n = 89)	24 weeks	Omega-3 index, omega-6 ratio, RBC EPA and DHA, liver fat	Increased omega-3 index, RBC EPA and DHA, decreased omega-6 ratio (p < 0.0001). Significant reduction in liver fat content in both groups	High-concentrate omega-3 supplementation significantly corrected omega-3 deficiency and reduced liver fat content in NAFLD patients

Effect of Omega-3 Polyunsaturated Fatty Acids on Liver Fat

**Figure 2:**
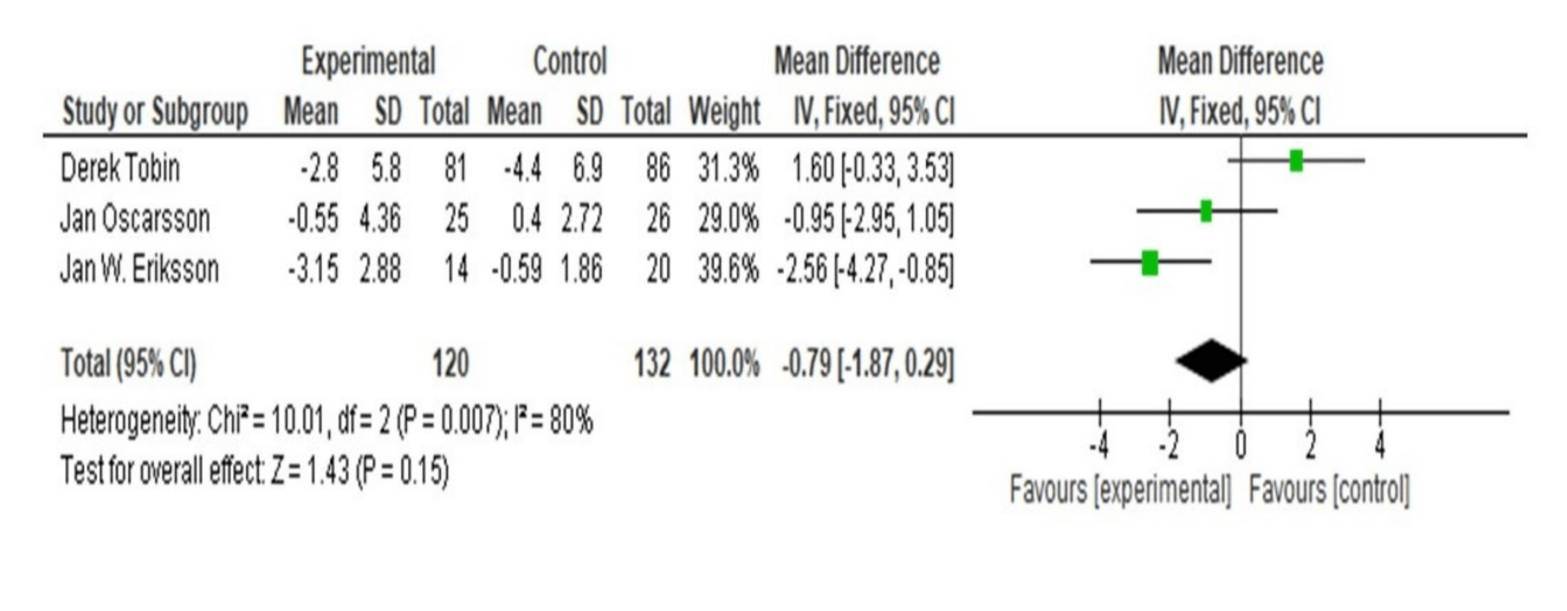
Forest plot of mean ± standard deviation and random effect model for the effect of omega-3 supplement in PDFF. [22,26,33]. PDFF: proton density fat fraction

The impact of omega-3 supplementation on liver proton density fat fraction (PDFF) was assessed using a pooled analysis of three trials. With a mean difference of 1.60 (95% confidence interval (CI) = -0.33, 3.53), omega-3 supplementation increased liver PDFF in the study by Tobin et al. research in a non-significant way [33]. The research conducted by Oscarsson et al. similarly revealed a non-significant decrease in liver PDFF, with a mean difference of -0.95 (95% CI = -2.95, 1.05) [22]. A significant decrease in liver PDFF was seen in the study by Eriksson et al., with a mean difference of -2.56 (95% CI = -4.27, -0.85) [26]. With omega-3 supplementation, there was a non-significant decrease in liver PDFF, as shown by the overall pooled mean difference of -0.79 (95% CI = -1.87, 0.29). The studies showed significant heterogeneity (I^2^ = 80%, p = 0.007, df = 2, Chi-square = 10.01). Omega-3 supplementation may not substantially lower liver PDFF overall, despite the considerable benefit shown in one of the individual trials, according to the test for the overall effect, which was not statistically significant (Z = 1.43, p = 0.15) (Figure 2).

Effect of n-3 Polyunsaturated Fatty Acids on Hepatic Enzyme Parameters

Several studies were conducted to assess the effect of n-3 PUFAs on hepatic enzyme parameters. In the study by Farhangi et al., the RDCSO group significantly outperformed the CSO group in terms of ALT and other liver tests [21]. Silymarin and *Phaleria macrocarpa* extract restored liver function indicators [27]. Spahis et al. discovered that omega-3 PUFA supplementation decreased ALT and the ALT/AST ratio, while NAFLD patients’ ALP levels were lowered by n-3 PUFA supplementation [29,30].

**Figure 3:**
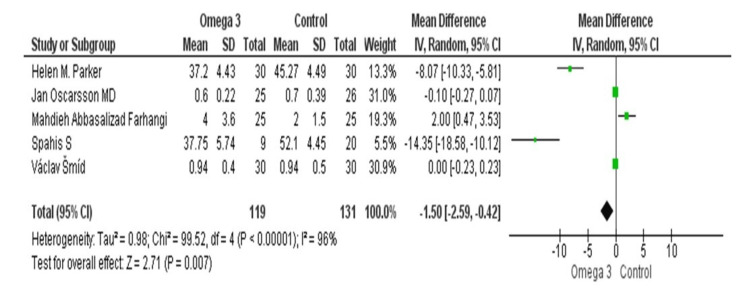
Forest plot of mean ±standard deviation and random effect model for the effect of n-3 PUFAs on AST. [21,22,25,29,31]. PUFA: polyunsaturated fatty acid; AST: aspartate aminotransferase

Using information from five trials, a pooled analysis was done to determine how omega-3 supplementation affected the levels of the AST enzyme. AST levels were significantly lower, with a mean difference of -8.07 (95% CI = -10.33, -5.81) [25]. The omega-3 supplementation in the study by Oscarsson et al. increased AST levels with a mean difference of 2.00 (95% CI = 0.47, 3.53) [22]. With a mean difference of -0.10 (95% CI = -0.27, 0.07), Farhangi et al. found a slight, non-significant decrease in AST levels [21]. In the study by Spahis et al., AST levels were shown to have significantly decreased, with a mean difference of -14.35 (95% CI = -18.58, -10.12) [29]. With a mean difference of 0.00 (95% CI = -0.23, 0.23), Šmíd et al. found no evidence of a significant change in AST levels [31]. With omega-3 supplementation, there was a substantial decrease in AST levels, as shown by the total pooled mean difference of -1.50 (95% CI = -2.59, -0.42). The studies exhibited a significant degree of heterogeneity (Tau² = 0.98, chi-square = 99.52, df = 4, p < 0.00001, I^2^ = 96%). The AST levels were significantly lower, according to the test for the overall impact (Z = 2.71, p = 0.007) (Figure 3).

**Figure 4:**
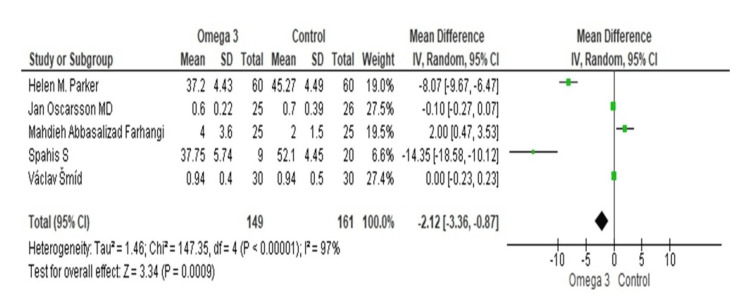
Forest plot of mean ±standard deviation and random effect model for the effect of n-3 PUFAs on ALT. [21,22,25,29,31]. PUFA: polyunsaturated fatty acid; ALT: alanine aminotransferase

To assess the impact of omega-3 supplementation on ALT enzyme levels across five trials, a pooled analysis was performed. Omega-3 supplementation significantly decreased ALT levels in the study by Parker et al., with a mean difference of -8.07 (95% CI = -9.67, -6.47) [25]. A small and non-significant decrease in ALT levels was reported by Farhangi et al., with a mean difference of -0.10 (95% CI = -0.27, 0.07) [21]. The study by Spahis et al. revealed a significant reduction in ALT levels, with a mean difference of -14.35 (95% CI = -18.58, -10.12) [29]. In the study by Oscarsson et al., there was a mean difference of 2.00 (95% CI = 0.47, 3.53) in ALT levels [22]. In the study by Šmíd et al., the mean difference in ALT levels was found to be 0.00 (95% CI = -0.23, 0.23), indicating no significant change [31]. The total pooled mean change was -2.12 (95% CI = -3.36, -0.87), indicating that omega-3 supplementation significantly decreased the levels of ALT. There was a significant amount of heterogeneity among the trials (Tau^2^ = 1.46, chi-square = 147.35, df = 4, p < 0.00001, I^2^ = 97%). A substantial decrease in ALT levels was shown by the test for the overall impact (Z = 3.34, p = 0.0009) (Figure 4).

**Figure 5:**
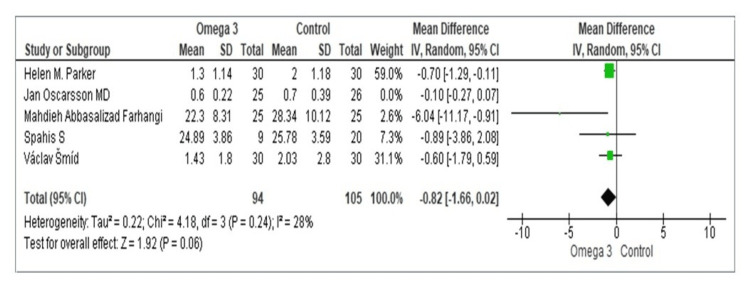
Forest plot of mean ± standard deviation and random effect model for the effect of n-3 PUFAs on GGT. [21,22,25,29,31]. PUFA: polyunsaturated fatty acid; GGT: gamma-glutamyl transferase

Using information from four investigations, a pooled analysis was performed to determine how omega-3 supplementation affected the levels of GGT enzymes. GGT levels were much lower, as shown by Parker et al., with a mean difference of -0.70 (95% CI = -1.29, -0.11) [25]. A small, non-significant drop in GGT levels was reported by Farhangi et al., with a mean difference of -0.10 (95% CI = -0.27, 0.07) [21]. GGT levels were shown to have significantly decreased in the study by Spahis et al., with a mean difference of -6.04 (95% CI = -11.17, -0.91) [29]. GGT levels did not significantly alter, according to Šmíd et al., with a mean difference of -0.60 (95% CI = -1.79, 0.59) [31]. The total pooled mean difference was -0.82 (95% CI = -1.66, 0.02), which suggests that omega-3 supplementation did not significantly lower GGT levels. There was little variation among the investigations (Tau^2^ = 0.22, chi-square = 4.18, df = 3, p = 0.24, I^2^ = 28%). While not statistically significant, the test for the overall impact neared significance (Z = 1.92, p = 0.06) and indicated a tendency toward lower GGT levels with omega-3 supplementation (Figure 5).

Effect of n-3 Polyunsaturated Fatty Acids on Serum Lipid Profiles

In numerous studies, omega-3 PUFAs had a substantial impact on blood lipid profiles. According to Farhangi et al., the RDCSO group’s lipid profile significantly improved compared to the CSO group [21]. According to Šmíd et al., the plasma lipid profile was enhanced (p < 0.005) by n-3 PUFA therapy [31]. According to a study by Musazadeh et al., supplementing with CSO dramatically reduced triglycerides, LDL-C, TC, and the atherogenic index [32]. Spahis et al. showed that omega-3 PUFA supplementation improved the lipid profile, while Song et al. reported that the PS + FO group’s TC and TG decreased by 11.57% and 9.55%, respectively [24,29].

Effect of n-3 Polyunsaturated Fatty Acids on Fasting Blood Sugar and Insulin Resistance

A few studies examined how n-3 PUFAs affected insulin resistance and fasting blood sugar levels. Supplementing with omega-3 boosted IL-6 and beta-cell activity considerably while having little impact on other insulin resistance and glycemic measures [19]. When dapagliflozin and omega-3 carboxylic acids are combined, Eriksson et al. found that the combination lowers hepatocyte damage biomarkers and fibroblast growth factor 21, suggesting a disease-modifying impact in NAFLD [26].

Effect of n-3 Polyunsaturated Fatty Acids on Body Mass Index

Studies have examined how n-3 PUFAs affect BMI. In comparison to the CSO group, Farhangi et al. found that the RDCSO group had significantly improved BMI and other anthropometric indicators [20]. According to Musazadeh et al., CSO supplementation dramatically increased the weight, BMI, and waist circumference of NAFLD patients, indicating that omega-3 fatty acids have a positive impact on both body composition and weight [31].

Quality Assessment

**Figure 6:**
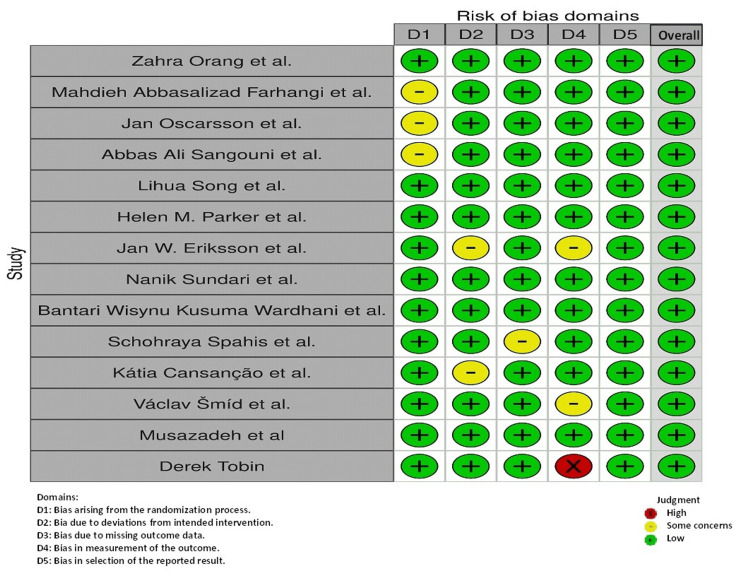
Quality assessment. [20-33].

The quality assessment of RCTs using the RoB 2.0 tool indicates that most studies exhibit a low risk of bias across all five domains, i.e., randomization process, deviations from intended interventions, missing outcome data, measurement of the outcome, and selection of the reported result. Specifically, the majority of studies show low risk, suggesting robust methodologies. However, some concerns are noted in the studies by Eriksson et al. [26], Parker et al. [25], Cansanção et al. [30], and Spahis et al. [29], primarily regarding missing outcome data and measurement of the outcome. Only one study, Musazadeh et al., demonstrates a high risk of bias in the measurement of the outcome [32]. Overall, the evidence from these RCTs is considered reliable and supports the validity of the meta-analysis conclusions (Figure 6).

Discussion

To evaluate the accuracy and effectiveness of omega-3 fatty acids in treating NAFLD, a systematic review and meta-analysis were conducted. The review updated the data on the therapeutic advantages of various therapies by including new research.

The findings of the meta-analysis on the impact of omega-3 PUFAs on liver fat were inconsistent. According to Vell et al., taking omega-3 supplements significantly lowers the risk of liver disease, indicating a potential preventive impact [19]. On the other hand, a drop in liver PDFF that was not statistically significant was seen in pooled data from investigations [22,26,33]. The observed discrepancy might potentially be explained by variations in the research design, demographic characteristics, dose, and duration of omega-3 supplementation. The pooled study failed to reveal a substantial overall impact, even though individual investigations, such as the one by Eriksson et al., showed considerable decreases in liver fat. These results emphasize the need for more extensive, well-planned research to elucidate the effect of omega-3 polyunsaturated fats on liver lipid composition in individuals with NAFLD.

Omega-3 supplementation was shown to significantly reduce the levels of liver enzymes, including ALT, GGT, and AST, according to a pooled study. For both AST and ALT, the total pooled mean difference was -1.50 (95% CI = -2.59, -0.42) and -2.12 (95% CI = -3.36, -0.87), respectively, demonstrating substantial improvements. However, these analyses showed a significant degree of heterogeneity (AST: I^2^ = 96%, ALT: I^2^ = 97%), indicating that the outcomes differed greatly among the trials. Even yet, the steady upward trend in liver enzymes points to the possibility of using omega-3 PUFAs as a helpful liver-healthy strategy. All of this is consistent with the earlier Yu et al.’s meta-analysis of RCT, except ALT. However, in the prior meta-analysis, as the ALT impact showed little heterogeneity, a fixed-effect model was used [34]. In addition, in the study by Huang et al., omega-3 PUFA demonstrated a potential trend toward improving AST, GGT, and LDL-C levels. The observed reductions in AST (IV 95% CI = -6.89 (-17.71 to 3.92), p = 0.21), GGT (IV 95% CI = -8.28 (-18.38 to 1.83), p = 0.11), and LDL-C (IV 95% CI = -7.13 (-14.26 to 0.0), p = 0.05) suggest a potentially beneficial effect of omega-3 PUFA on these markers.

Several studies have shown the considerable improvement of blood lipid profiles with omega-3 PUFAs. The investigations revealed notable increases in HDL-C and significant decreases in TGs, TC, and LDL-C [24,32]. Due to the frequent comorbidity of dyslipidemia, these lipid-lowering benefits are especially significant in NAFLD patients. Better lipid profiles may reduce the risk of NAFLD developing into more serious liver disorders and improve general cardiovascular health. It was also shown in a prior meta-analysis, which discovered that supplementing with n-3 PUFAs considerably raises BMI, TC, TG, and HDL plasma levels in fatty liver patients [17]. Whereas, a meta-analysis of 14 RCTs, encompassing 15 data sets, demonstrated a significant overall reduction in TG levels following omega-3 supplementation (WMD = -15.71 mg/dL, 95% CI = -25.76 to -5.65, p = 0.002). However, this effect was accompanied by substantial heterogeneity (I^2^ = 88.3%, p < 0.001). Subgroup analyses provided further insights, revealing that the reduction in TG was significant primarily in studies involving participants aged 13 years or younger (WMD = -25.09, 95% CI = -43.29 to -6.90, p = 0.007), (I^2^ = 84.6%, p < 0.001) and those with hypertriglyceridemia (WMD = -28.26, 95% CI = -39.12 to -17.41, p < 0.001), (I^2^ = 0.0%, p = 0.934). Conversely, omega-3 supplementation did not produce significant changes in total cholesterol, HDL, or LDL levels. Notably, a non-linear analysis indicated that the duration of treatment had a significant impact on HDL levels (non-linearity = 0.047), suggesting that the length of supplementation may be an important factor in modulating HDL outcomes [35].

While some studies have suggested that omega-3 supplementation may lead to substantial changes in BMI and other anthropometric indices, the current meta-analysis did not find evidence supporting these effects [21,32]. Specifically, omega-3 fatty acid supplementation showed no significant impact on reducing body weight (SMD = -0.00, 95% CI = -0.26 to 0.25), BMI (SMD = -0.07, 95% CI = -0.32 to 0.17), or waist circumference (SMD = -0.16, 95% CI = -0.51 to 0.19). These findings suggest that, despite its potential benefits for weight control and liver function in the context of NAFLD management, omega-3 supplementation does not appear to significantly alter anthropometric indices in children and adolescents. Given the importance of weight reduction and decreased adiposity in NAFLD management, further large-scale studies with larger sample sizes are needed to clarify the effects of omega-3 fatty acids on these outcomes in younger populations. Notably, animal models have demonstrated significant decreases in liver enzymes and markers of liver injury and fibrosis with omega-3 supplementation, indicating a potential area for further exploration [27,28,36].

Limitations

Significant study heterogeneity, differences in intervention procedures and assessment techniques, brief study durations, and possible publication bias were some of the constraints in this meta-analysis. Determining the ideal doses and formulations was made more difficult by these considerations, which also made the conclusions less generalizable. Longer-term clinical trials, standardizing measurement methods, investigating ideal dosages, incorporating a variety of populations, evaluating combination therapies, mitigating publication bias, and examining mechanistic insights should be the focus of future research to improve our understanding of and ability to treat NAFLD with omega-3 PUFAs.

## Conclusions

This systematic review and meta-analysis provides new insights into the effectiveness of omega-3 polyunsaturated fats in the treatment of NAFLD. While the impact on liver fat content is still inconclusive, omega-3 PUFAs show beneficial effects on liver enzymes, serum lipid profiles, and anthropometric indices. Though further investigation is needed, omega-3 supplementation may offer NAFLD patients a valuable adjuvant treatment, enhancing metabolic profiles and liver function. Future studies should focus on standardizing doses, understanding patient-specific responses, and exploring the synergistic effects of omega-3s with other treatments and lifestyle changes.
